# Severe complications in acute Epstein Barr infection – case reports

**DOI:** 10.1186/1471-2334-13-S1-P104

**Published:** 2013-12-16

**Authors:** Anuța Bilaşco, Monica Luminos, Anca Drăgănescu, Gheorghiță Jugulete, Magda Vasile, Angelica Vişan, Cristina Negulescu, Cristina Popescu, Camelia Kouris, Mădălina Maria Merişescu, Georgeta Constantinescu, Endis Osman

**Affiliations:** 1 National Institute for Infectious Diseases “Prof. Dr. Matei Balş”, Bucharest, Romania; 2 Carol Davila University of Medicine and Pharmacy, Bucharest, Romania

## Background

Epstein-Barr virus (EBV) infects almost a large percent of the world’s adult population. Antibody prevalence rates reach 95% or higher among elderly individuals. Even in the pediatric population, Epstein Barr infection is common. Young children most likely acquire primary EBV infection from close contact that involves exchange of oral secretions via shared items such as toys, bottles and other objects. It may present with mild or no symptoms or with potentially life-threatening fulminant illness.

Infectious mononucleosis caused by Epstein-Barr virus (EBV) is usually a benign systemic viral disease and occurs without sequelae but occasionally, it may be complicated by a variety of neurologic, hematologic, respiratory and hepatic complications.

## Case reports

We present the cases of three children admitted in the Pediatric Intensive Care Unit of the National Institute for Infectious Diseases “Prof. Dr. Matei Balş” with serious complications associated to Epstein-Barr infection. The first case described by us is a 6 year-old girl diagnosed with acute encephalomyelitis, the second case a 2 year-old girl diagnosed with Guillain-Barré syndrome and the third case a 3 year-old girl with acquired hemophagocytic lymphohistiocytosis.

The diagnosis was based on clinical signs, hematological, biochemical and serological assays, MRI (in children with neurological conditions) and histological examination (in the patient with hematological complications). No evidence for immunodeficiency was detected in these children with acute infection.

Our patients required intensive care measures, antiviral therapy, corticosteroids, immunoglobulin therapy, physiotherapy (for neurological complications), blood product transfusions and immunosuppressive therapy (for hematologic complications).

The evolution was favorable with complete recovery for all 3 children.

## Conclusion

Although Epstein Barr infection often involves asymptomatic or mild disease, severe complications can occur even in immunocompetent children, with potentially fatal outcome. With appropriate therapy a good outcome occurred in all our patients reported with successful neurological and hematological recovery.

The biology of Epstein Barr virus, virus-host interaction and the human immune response to infection are still genuine challenges for medicine today.

